# The Functional Characteristics of Goat Cheese Microbiota from a One-Health Perspective

**DOI:** 10.3390/ijms232214131

**Published:** 2022-11-16

**Authors:** Bruno Tilocca, Alessio Soggiu, Federica Iavarone, Viviana Greco, Lorenza Putignani, Maria Vittoria Ristori, Gabriele Macari, Anna Antonella Spina, Valeria Maria Morittu, Carlotta Ceniti, Cristian Piras, Luigi Bonizzi, Domenico Britti, Andrea Urbani, Daniel Figeys, Paola Roncada

**Affiliations:** 1 Department of Health Sciences, University ‘Magna Græcia’ of Catanzaro, Viale Europa, 88100 Catanzaro, Italy; 2 One Health Unit, Department of Biomedical, Surgical and Dental Sciences, University of Milano, Via della Commenda 10, 20133 Milano, Italy; 3 Department of Basic Biotechnological Sciences, Intensivological and Perioperative Clinics, Catholic University of Sacred Heart, Largo Vito, 00168 Rome, Italy; 4 Clinical Chemistry, Biochemistry and Molecular Biology Operations (UOC), Agostino Gemelli Foundation University Hospital IRCCS, Largo Agostino Gemelli 8, 00168 Rome, Italy; 5 Unit of Parasitology, Unit of Human Microbiome, Bambino Gesù Children’s Hospital IRCCS, Piazza Sant’Onofrio, 4, 00165 Rome, Italy; 6 GenomeUp SRL, Viale Pasteur, 6, 00144 Rome, Italy; 7 Ottawa Institute of Systems Biology, University of Ottawa, 451 Smyth Road, Ottawa, ON K1H 8M5, Canada

**Keywords:** goat cheese microbiota, one health, metaproteomics, targeted metagenomics, cheese microbiota, raw milk, animal infectious disease

## Abstract

Goat cheese is an important element of the Mediterranean diet, appreciated for its health-promoting features and unique taste. A pivotal role in the development of these characteristics is attributed to the microbiota and its continuous remodeling over space and time. Nevertheless, no thorough study of the cheese-associated microbiota using two metaomics approaches has previously been conducted. Here, we employed 16S rRNA gene sequencing and metaproteomics to explore the microbiota of a typical raw goat milk cheese at various ripening timepoints and depths of the cheese wheel. The 16S rRNA gene-sequencing and metaproteomics results described a stable microbiota ecology across the selected ripening timepoints, providing evidence for the microbiologically driven fermentation of goat milk products. The important features of the microbiota harbored on the surface and in the core of the cheese mass were highlighted in both compositional and functional terms. We observed the rind microbiota struggling to maintain the biosafety of the cheese through competition mechanisms and/or by preventing the colonization of the cheese by pathobionts of animal or environmental origin. The core microbiota was focused on other biochemical processes, supporting its role in the development of both the health benefits and the pleasant gustatory nuances of goat cheese.

## 1. Introduction

Goat cheese is a key element in the Mediterranean diet and is among the most frequently consumed dairy products globally. The texture, flavor, and organoleptic properties of the cheese depend on several factors, including (but not limited to) the cheesemaking process, the animal breed, and the breeding management. Emerging evidence underlines the pivotal role of the microbiota, and its continuous evolution, in the conditioning of a cheese’s characteristics. The complex microbial diversity harbored in the milk converts its components, mainly carbohydrates and proteins, into secondary products and/or substrates that further trigger the growth and metabolism of microorganisms. This results in the continuous restructuring of the microbiota and the accumulation of myriads of molecules of microbial origin constituting the cheese mass, such as fatty acids, volatile organic compounds (VOCs), amines, ketones, free amino acids, phenols, alcohols, aldehydes, lactones, and sulfur compounds [1,2,3,4]. On the other hand, the presence of harmful elements, either of animal or environmental origin, poses health and hygiene problems. As of today, most commercial and large-scale cheese factories carry out milk treatment procedures (e.g., thermization and pressurization) to standardize the milk quality and drastically reduce the milk’s microbial load and diversity [5]. In contrast, the great majority of traditional dairy products are still produced using raw milk [6], thus benefitting from a high level of microbial biodiversity operating across the stages of the cheesemaking process. Nevertheless, higher hygienic standards and precautions are required throughout the whole production process [6,7].

Milk and cheese act as the “point of contact” between animal, human, and environmental health; therefore, the accurate assessment of the microbiota contained in these products is an important tool of One-Health relevance, besides being of great importance in other respects, such as biosafety, technological processing, and nutritional and nutraceutical value. The rapid advance of the metaomics discipline has enabled the detailed characterization of the microbial communities harbored by virtually all ecological niches. Metagenomic and metabolomic investigations are the most commonly employed approaches in cheese microbiota research, providing information on the composition and genetic potential of the sampled microbial community along with the overall array of metabolites produced by the consortia of microorganisms associated with cheese. The supplementation of this information with metaproteomics is desirable, as it would provide valuable information on the protein repertoire and the biochemical pathways being effectively implemented by the microbiota components under the sampling conditions. Nevertheless, metaproteomic investigations of milk by-products are rare owing to the technical difficulties that prevent the common adoption of the metaproteomics approach in the characterization of cheese microbiota. To the best of our knowledge, only a few studies available in the literature have employed metaproteomics for the investigation of cheese microbiota [8,9,10], and no metaproteomics studies are available on raw goat milk cheese.

Considering the potential of studying cheese microbiota and the paucity of cheese metaproteomics studies, we conducted 16S rRNA gene sequencing and a metaproteomics study to obtain a comprehensive picture of a typical raw goat cheese microbiota in terms of both composition and activity. This is the first metaomics-based study of a typical raw goat milk cheese investigating the microbial community dynamics on the rind and in the core of the cheese wheel across different ripening periods. Moreover, we provide insights into the microbial interactions occurring among the naïve and environmental bacteria and biochemical strategies for guaranteeing the biosafety of typical raw milk by-products.

## 2. Results

### 2.1. Metataxonomic Analysis of the Microbiota

The sequencing of the V3–V4 regions of the 16S rRNA gene identified an average of 27,500 reads per sample (20,000–35,000 reads). The median Good’s coverage of approximately 0.985 for both the rind and core sample groups (Additional File S1) was indicative of a satisfactory nucleic acid extraction performance, and only a minor part of the 16S-based information was neglected in the present approach. Analogous analytical evaluations were conducted for the samples labeled on a ripening-timepoint basis (Additional File S1). Sequencing reads were quality-filtered and trimmed before being binned into ASVs.

**Figure 1:**
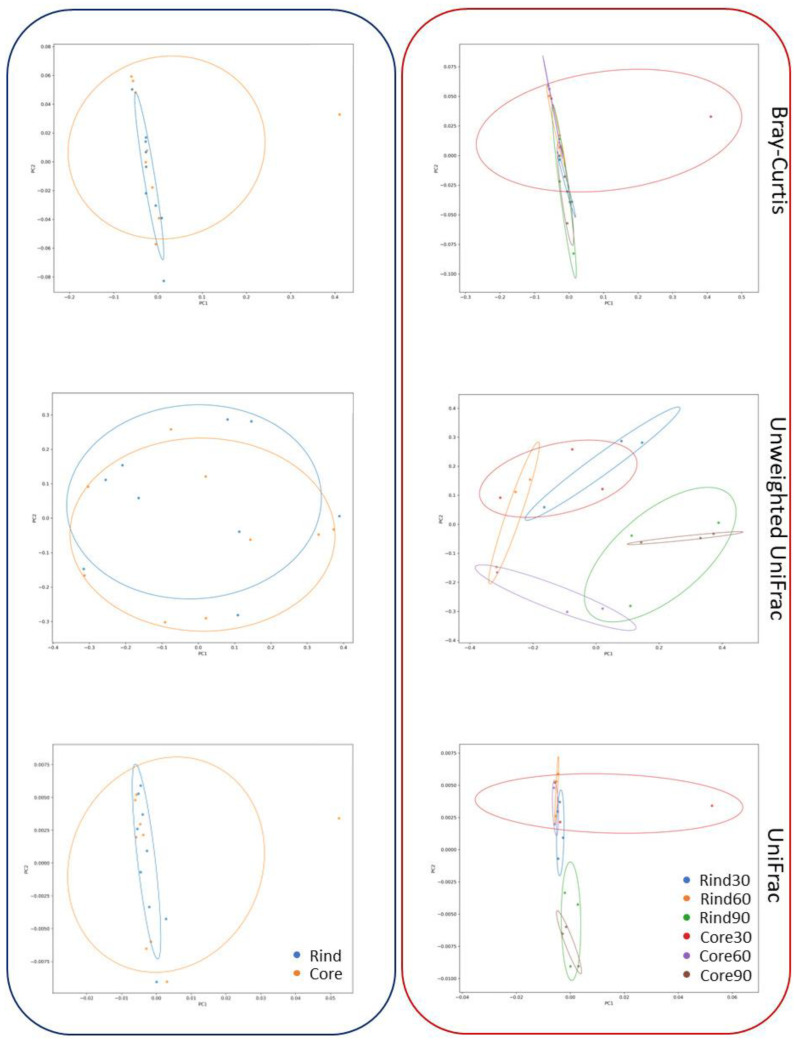
PCO plots of the 16S rRNA gene-sequencing data. The overall DNA-based dataset is visualized as PCO plots according to Bray–Curtis similarity, unweighted UniFrac, and UniFrac. Blue framed plots depict samples sorted according to the cheese-wheel depth (i.e., rind or core). Red-framed plots depict samples stratified according to both the cheese-wheel depth and ripening timepoint.

Data from the DNA-based investigations were assembled in data matrices according to the Bray–Curtis similarity and the weighted and unweighted UniFrac, as depicted in the PCO plots in Figure 1. Here, the sample ordination produced a scattered pattern of 16S rRNA profiles, preventing a clear distinction between the microbial communities harbored in the rind and the core samples, as well as between the microbiota at different cheese ripening stages (*p* > 0.05).

**Figure 2:**
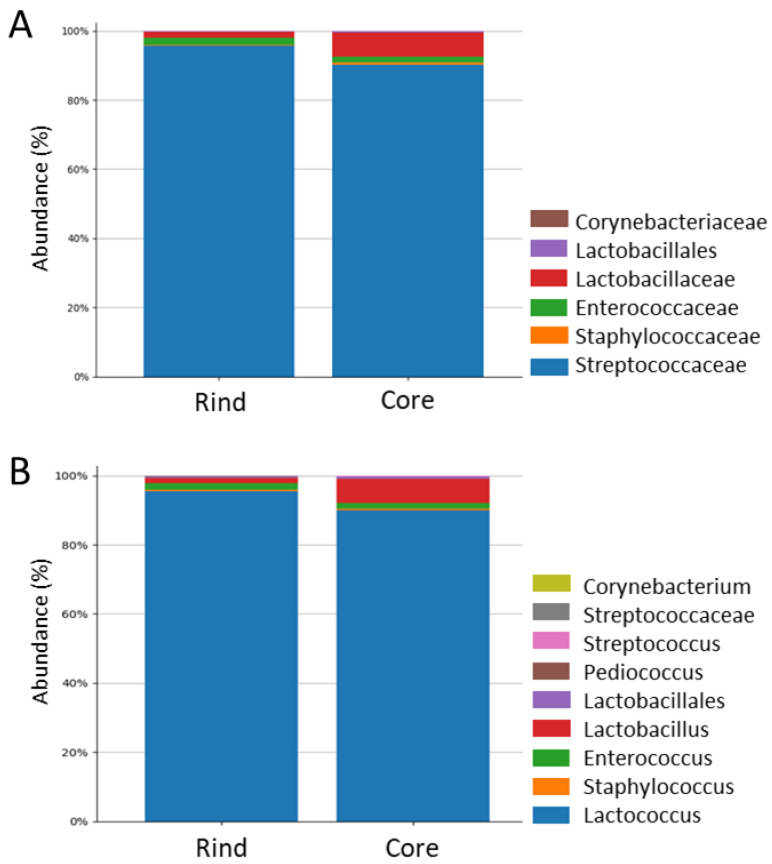
Microbiota composition assessment by 16S rRNA gene sequencing. The bar charts visualize the composition of the microbial community harbored in the rind and core of the cheese wheel regardless of the ripening timepoint. (**A**) Microbiota composition at the family taxon level; (**B**) microbiota composition at the genus level. Higher taxonomic levels are displayed in both (**A**,**B**) when it was not possible to attribute a family or genus to the OTU, respectively.

The identified ASVs, sorted on a sample-label basis, portrayed a stable microbiota composition across the three ripening periods, as supported by ANOVA (30, 60, and 90 ripening days, *p* > 0.05). On the other hand, the sorting of the OTUs based on the cheese-wheel depth (i.e., core or rind) revealed tendentially different microbiota compositions at the taxonomic levels of family and genus (Figure 2), with the genus Lactobacillus and family Lactobacillaceae being overrepresented in the core-associated microbiota, thus driving the structural alterations evidenced by the comparative evaluation of the microbial communities.

The taxonomic composition of the microbial communities was further employed as the input for the assessment of the functional potential of the microbiota using PICRUST analysis (Additional File S2). Altogether, no large differences were found in the comparative evaluation of the functional potential of the microbiota colonizing the rind and the core regions, as well as among the microbial communities at the different ripening timepoints. These observations were statistically supported, as shown by the resemblance matrices drawn according to Bray–Curtis similarity and the Euclidean distances of the microbial communities’ functional profiles (Additional File S3).

### 2.2. Metaproteomics Investigation of the Microbial Communities

**Figure 3:**
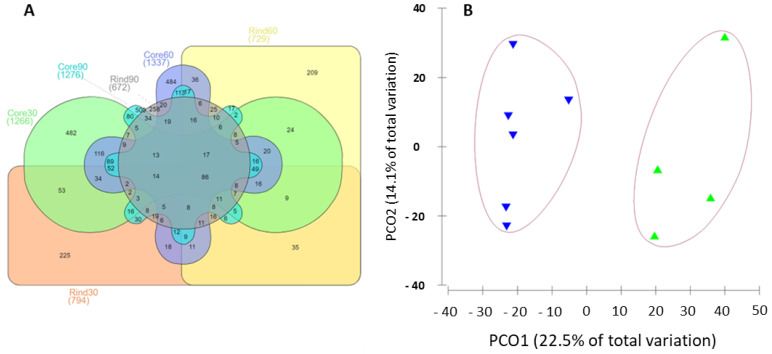
Metaproteomics dataset. (**A**) The identified proteins. The Venn diagram displays the number of proteins identified in each of the sample groups taken into consideration in the present study. Specifically, the diagram shows the size of the metaproteome identified in the rind and core samples at the ripening timepoints of 30, 60, and 90 days. (**B**) The ordination of the dataset in a PCO plot, highlighting the clear separation of the rind and core samples according to their identified protein repertoires.

The investigation of the microbiota using metaproteomics relied on the identification of approximately 3500 proteins among the samples extracted for different ripening timepoints and cheese-wheel depths. A variable number of proteins was identified in each of the sample groups considered in the study, with as many as 482, 500, and 484 unique proteins for the core samples at 30, 60, and 90 days of ripening, respectively, whilst 225, 209, and 258 unique proteins were identified in the rind samples at 30, 60, and 90 days of ageing. Nevertheless, most of the identified proteins were shared between pairs of sample groups in diverse combinations, as depicted in Figure 3, panel A. In line with the DNA-based investigations, the visualization of the metaproteomic dataset as a PCO plot revealed a scattered distribution of the samples as a function of the ripening period. Additionally, arranging the samples according to the cheese-wheel depth resulted in a clear separation of the metaproteomic profiles along the PCO1 axis (Figure 3, panel B). The clustering of the rind and core samples was also supported by the ANOVA statistical test, with *p* < 0.01.

**Figure 4:**
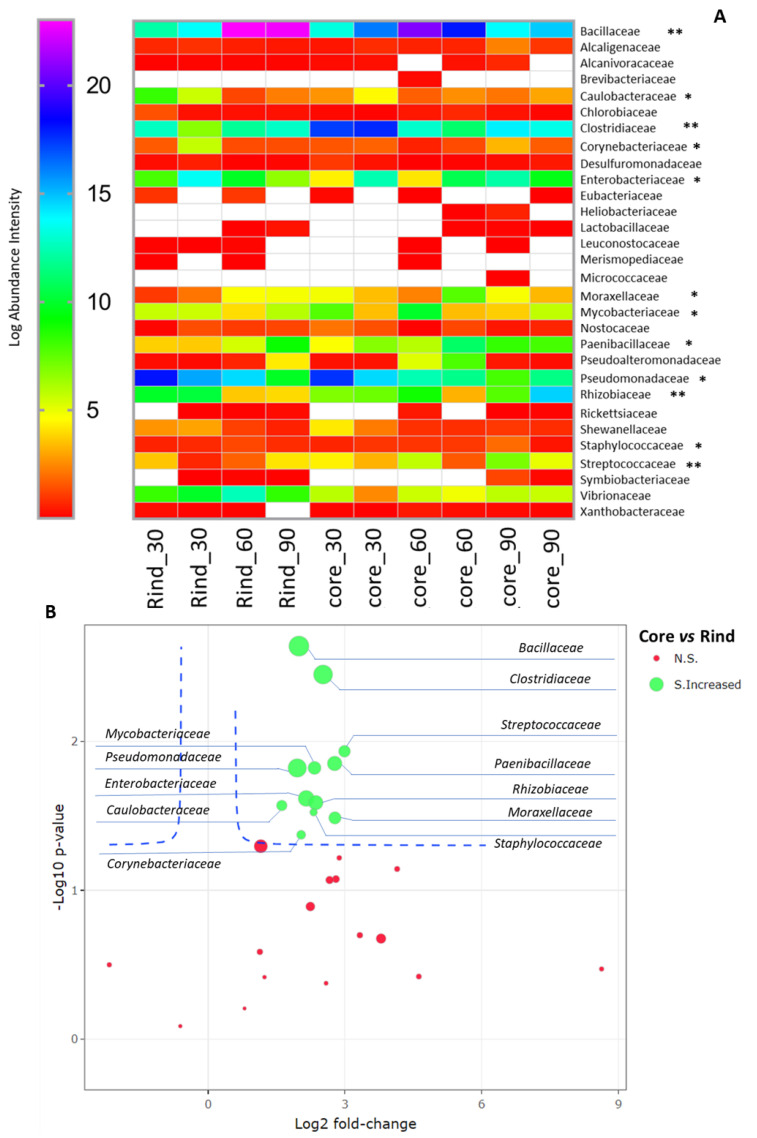
Microbiota composition based on the identified protein repertoire. (**A**) Quantitative composition of the microbial communities harbored in the rind and core of the cheese wheel at 30, 60, and 90 days of ripening. T-tests on a sample group basis (i.e., rind and core) were performed to highlight the contributors to the statistically significant differences in the structure of the microbiota between the rind and the core. Single and double asterisks indicate *p* < 0.05 and *p* < 0.01, respectively. (**B**) Volcano plot summarizing the bacterial families significantly overrepresented in the core samples (green circles) according to the T-test analysis. Non-statistically significant bacterial families are represented by red circles. The average intensity of each bacterial family is indicated by the diameter of its circle.

The taxonomic composition of the microbial communities determined by metaproteomics suggested a higher bacterial diversity, at the family level, when compared with the companion DNA-based approach. An overview of the diversity indexes calculated for the microbial communities over the diverse cheese-wheel depths and ripening timepoints is provided in Additional File S4. The cumulative abundance of the proteins relative to each bacterial family revealed differences (ANOVA, *p* < 0.05) in the composition of the rind and core microbiotas. On the other hand, the overall microbiota architecture at the diverse ripening timepoints was stable, as assessed by both the ANOVA and the pairwise PERMANOVA statistical tests (*p* > 0.05). Considering the above observations, we focused on the rind and core microbiota to define the bacterial families driving the differences in the taxonomic structures of these microbial communities. Regardless of the ripening timepoints, proteins belonging to the families Bacillaceae, Rhizobiaceae, Clostridiaceae, Streptococcaceae, Caulobacteriaceae, Enterobacteriaceae, Moraxellaceae, Mycobacteriaceae, Paenibacillaceae, Pseudomonadaceae, and Staphylococcaceae were overrepresented in the core samples (*p* < 0.05), as reported in the volcano plot of Figure 4. Similarly, the evaluation of the protein abundance profiles by least common ancestor according to linear discriminant analysis (LDA) identified Paenibacillaceae and Vibrionaceae as the major discriminating families, with the former being overrepresented in the core samples and the latter being more abundant in the rind microbiota (Additional File S5).

**Figure 5:**
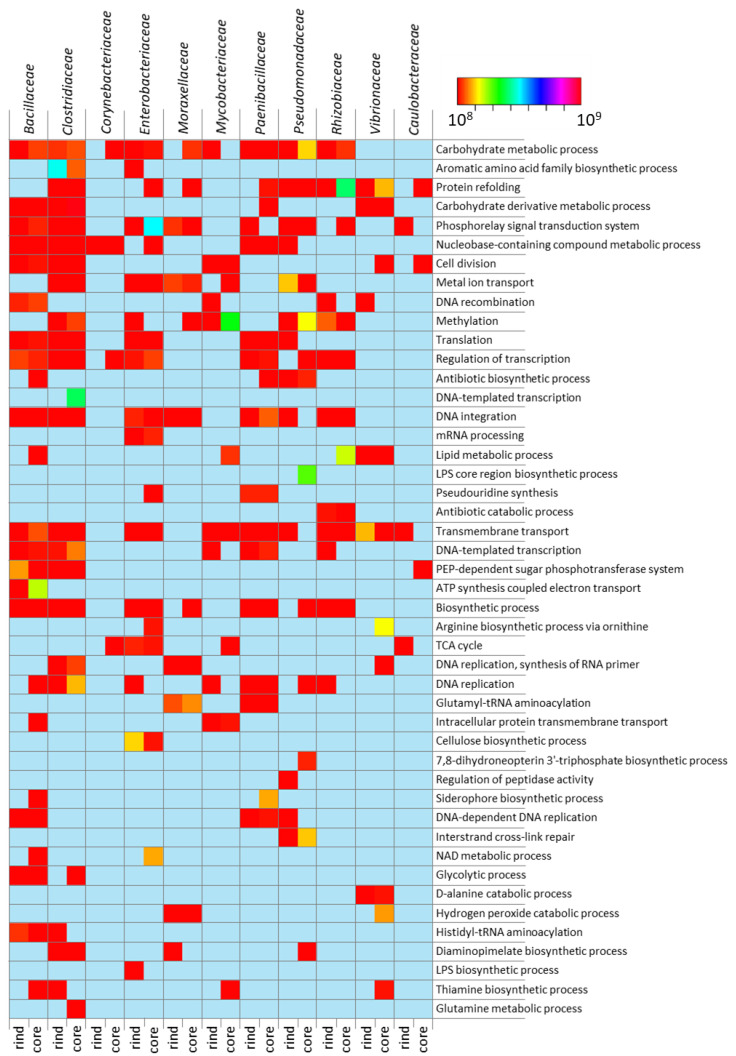
Functional characterization of the rind and core microbiota. The heatmap displays the biological processes participated in by the microbiota of the core and rind. The figure details the functional concerns of the bacterial families whose cumulative protein abundance was above the pre-fixed threshold of 1% total protein abundance. The color scale is relative to the protein abundance intensity.

The functional characterization of the cheese microbiota was accomplished by sorting the identified protein repertoire using a variety of protein ontology data repositories, such as Gene Ontology (GO), PFAM, and TIGRFAM. A detailed overview of the functional classification of the identified protein repertoire is provided in Additional File S6. Altogether, the functional classification revealed differences in the activities of the rind- and core-associated microbiota (*p* < 0.01) according to the functional classification data repositories. Besides ANOVA, further statistical evaluations underlined the “blind” grouping of the microbial communities harbored in the rind and core of the cheese wheel according to their different functional profiles. In line with the previous observations, no statically significant differences were observed between microbial communities at the selected ripening timepoints (*p* > 0.05) (Additional File S7). Protein sorting based on the GO biological processes provided an overview of the major functional concerns of the microbiota in the rind and core samples. The rind-associated microbiota was intensively involved in the “antibiotic catabolic process”, the “cellulose biosynthetic process”, the “glutamine metabolic process”, and “histidyl-tRNA aminoacylation” as compared with its core counterpart. On the other hand, the core microbiota was exclusively involved in biological processes such as “polyketide metabolism” and “siderophore biosynthesis”. Additionally, the core microbiota was much more involved in biological processes such as “carbohydrate derivative metabolism”, “cell division”, and the “phosphorelay signal transduction system” (Figure 5). A deeper investigation of the functional data elucidated the bacterial families principally involved in the biological processes peculiar to each microbiota. In the rind microbiota, the family Rhizobiaceae was the main player in the “antibiotic catabolic process”, suggesting that bacterial organisms of environmental origin were the principal target of the antibiotic-based defense. The “cellulose biosynthetic process” was led by the Enterobacteriaceae, whereas the “histidyl-tRNA aminoacylation” biological process was accomplished by the Bacillaceae and Clostridiaceae (Figure 5).

The functional characterization of the core microbiota highlighted the families Paenibacillaceae and Bacillaceae as the major contributors to the “antibiotic biosynthetic process” and “siderophore biosynthesis”, suggesting the role of these organisms in maintaining food biosafety by outcompeting pathobionts. Additionally, the role of the core microbiota in developing the cheese’s organoleptic properties was primarily performed by Bacillaceae and Rhizobiaceae, which were exclusively involved in the “lipid metabolic process”, whereas “arginine biosynthesis” was mostly accomplished by Vibrionaceae and Enterobacteriaceae. Additionally, members of the family Pseudomonadaceae were the major contributors to the “7,8-dihydroneopterin 3-triphosphate biosynthetic process” (Figure 5).

Considering the dynamic structure and functions of the microbial communities in the different cheese-wheel regions, the quantitative metaproteome was used to elucidate the correlation network between the bacterial families in the cheese core and rind samples.

**Figure 6:**
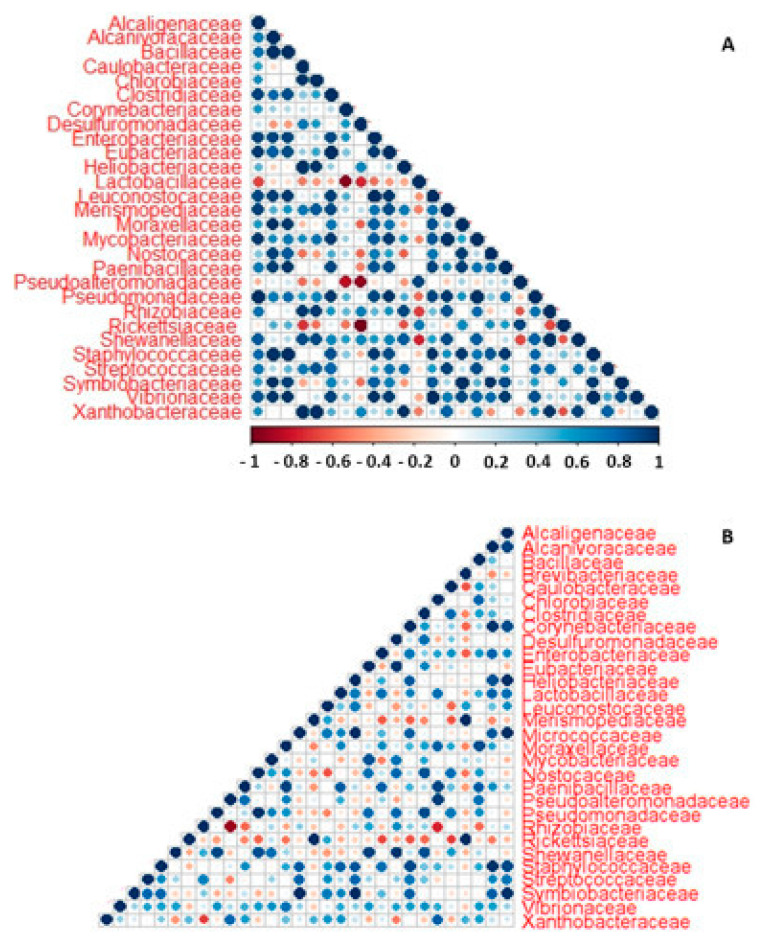
Correlogram analysis. The correlations computed among the members of the microbial communities harbored in the rind and core of the cheese wheel are depicted in panels (**A**,**B**), respectively.

Interestingly, the bacterial families Enterobacteriaceae, Rhizobiaceae, Bacillaceae, and Clostridiaceae showed a strong positive correlation with each other, supporting the previous observations regarding both the structural and functional makeup of the microbial community harbored on the surface region of the cheese wheel (Figure 6A).

Regarding the core microbiota, the correlogram analysis indicated that the family Paenibacillaceae was negatively correlated with most of the identified bacterial organisms of likely environmental origin (e.g., Desulfuromonadaceae, Mycobacteriaceae, and Ricketsiaceae), whilst it was positively correlated with other families such as Lactobacillaceae, Enterococcaceae, and Bacillaceae. The family Bacillaceae, in turn, supported the microbiota biodiversity by positively correlating with most of the bacterial families identified in the survey. In addition, the bacterial families identified as playing a role in the development of the cheese organoleptic properties (e.g., Bacillaceae, Rhizobiaceae, Vibrionaceae, and Enterobacteriaceae) were linked to each other by positive correlations, both strong and weak (Figure 6B).

## 3. Discussion

The microbial consortia of milk and its by-products are important bioindicators of animal health and the microbial exchange occurring through the human–animal–environment network. The fine orchestration of microbial metabolic functions is the foundation of the cheesemaking technological process, including the development of the gustatory and/or olfactive nuances peculiar to each cheese and the maintenance of the biosafety of dairy products. Most typical cheeses are made with raw, unprocessed milk carrying a high level of microbial diversity, whose importance is still largely debated. On the one hand, employing milk rich in microbial biodiversity enables the control of food biosafety along with the development of unique characteristics through the exploitation of the variable and versatile arrays of metabolic routes. Contrary to this, a higher level of microbial diversity might involve pathobionts and/or spoilage organisms; thus, a reduction in the naïve milk-associated microbial flora is thought to be key for guaranteeing the quality and biosafety of milk and its by-products [6,11]. Although both arguments are scientifically sound, the myriad of variables influencing both the structural and functional networks of the microbiota make the effect of the microbial consortia on various aspects of the cheesemaking process unpredictable. This prompted us to conduct a thorough investigation into each microbiota to elucidate how the microbial interconnections were shaped throughout the experimental duration.

In this study, we conducted the first metaomics survey of the microbiota associated with a traditional cheese made with raw goat milk. The cheese selected as the study model was Caprino Nicastrese, an artisanal goat cheese produced in Calabria, in the south of Italy. To the best of our knowledge, two studies have so far been performed implementing “pre-omics” approaches to evaluate the presence of selected bacterial specimens [12,13], but a comprehensive analysis of the typical goat cheese microbiota is lacking. The sampling strategy we adopted relied on our initial hypothesis that cheese regions with diverse physicochemical features (e.g., oxygen availability) would host different microbial communities, whose overall metabolism was likely pivotal to specific aspects of the cheese. The ripening timepoints selected in our study mirrored the variants of the cheese that are currently sold (i.e., Caprino Nicastrese ripened at 30, 60, and 90 days).

Both DNA and protein datasets depicted distinct microbiotas in the rind and core of the cheese wheel. The core-associated microbiota was characterized by the emergence of new bacterial families (e.g., *Brevibacteriaceae* and *Micrococcaceae*) along with the increased abundance of other bacterial families such as *Lactobacillaceae* and *Paenibacillaceae*. Altogether, this elucidates how the ecological niche (i.e., cheese rind or core) shapes the microbiota architecture and microbial metabolism to transform the dairy product and preserve it from spoilage. Our observations diverged from other descriptions of the microbiota of this cheese, which identify *Lactobacillus* spp. and *Enterococcus* spp. as the most abundant organisms [12,13]. These variations in microbiota composition were primarily attributed to the different investigation methods. Pino and colleagues [12] employed culture-dependent methods, which intrinsically overestimate the most common bacterial organisms at the expense of others. Additionally, farm-to-farm variability is to be expected due to the lack of strict production specifications and the changing environmental variables that influence the microbiota composition [12]. Moreover, this previous investigation did not distinguish between core and surface microbiota while assessing the microbial consortia composition. As the current study shows, the different cheese-wheel depths are associated with specific microbiota compositions and functions. Altogether, these factors mean that the studies are not easily comparable; rather, an integration and complementation of the outcomes should be considered.

Different pictures of the general microbiota composition were drawn by the 16S rRNA gene-sequencing and metaproteomics approaches. For instance, the family *Streptococcaceae* was identified as the most abundant in the core and rind microbiota by the DNA-based approach, whereas the most abundant protein profile belonged to the family *Bacillaceae,* regardless of the cheese-wheel depth. Additionally, the taxonomic assessment by metaproteomics identified a higher bacterial heterogeneity at the family level than 16S rRNA gene sequencing. The variations in the observations within the present study stemmed from the different principles these methods are based on. Each method targets different biological macromolecules and thus presents diverse technical drawbacks [14]. In addition, metaproteomics enables the identification of a higher level of bacterial complexity, since the changes in the abundance of expressed proteins are detected earlier than the changes in the number of DNA copies targeted by 16S rRNA gene sequencing [14,15,16,17].

Surprisingly, a stable microbiota composition was described by both 16S rRNA gene sequencing and metaproteomics in the samples stratified according to the ripening timepoint. This observation was unexpected. In light of this, we believe that the major structural rearrangements occurred in the early stages of the cheesemaking process (i.e., before 30 days, which was our earliest sampling timepoint), and that only minor reshaping took place after the “microbiologically driven” ripening stage, resulting in no statistically significant differences in the microbial consortia composition across the sampling timepoints. An alternative/complementary interpretation of this outcome would support the slow and continuous shaping of the microbiota, so that only a longer ripening window could highlight any statistically significant structural changes. This view is also supported by a recent study performed on Cheddar cheese made from raw milk. Here, the major shaping of the microbial consortia occurred in the very early stages of the cheese production, and a relatively stable microbiota composition was reported over the next 26 weeks of ageing [18]. Another recent study on raw goat cheese ripened for 5–15 days reported a stable microbiota composition, again supporting the notion of a slow-but-continuous shaping of the microbial consortia [19].

The functional characterization of the microbial communities was in agreement with the previous observations made by grouping the samples according to the cheese-wheel depth only. This clustering was entirely supported by the functional categorization of the protein repertoire, underlining the different functional concerns of the microbiota harbored in the rind and core of the cheese wheel.

The ontology of the rind protein repertoire identified a microbial community mostly involved in the maintenance of food biosafety by preventing the cheese surface from being colonized by foreign microbial organisms such as pathobionts or spoilage bacteria. This microbial consortium was, indeed, involved in “cellulose biosynthesis”, a biological process carried out by aerobic acetic acid bacteria that perform the oxidative fermentation of a variety of sugar substrates and, once they have exhausted the lactose as the main carbon source, produce cellulose as a by-product [20]. The bacterial cellulose produced on the rind surface “wraps” the dairy product, providing physical support and facilitating symbiotic interconnections among the microorganisms that preserve the food from colonization by external microorganisms [21]. In line with the above, the overexpression of the “antibiotic catabolic process” supported the occurrence of competition mechanisms between the naïve and environmental flora, suggesting that the bacterial families encoding for this biological process had experienced a chemical attack. Additionally, the increased abundance profile of proteins related to the “glutamine metabolic process” might have indicated the involvement of the rind microbiota in the biosynthesis of nitrogen-containing compounds, to which are attributed several physiological and technological functions, such as antimicrobial properties and the development of typical organoleptic features [22].

In comparison to the rind microbiota, the microbial community in the core of the cheese wheel was more heterogeneous, in both structural and functional terms. The bacterial involvement in the maintenance of product biosafety remained, although other biochemical routes were employed. In addition, the core microbiota seemed focused on more complex and diverse biological functions, ranging from the conservation of bacterial metabolism to the array of processes involved in the development of the so-called “added values” of typical cheeses in both nutraceutical and sensorial terms. The metabolic processes performed by the core microbiota indicated the greater participation of this microbial community in biological processes such as DNA replication, protein biosynthesis, and cellular respiration. The latter is interestingly represented by the case of H_2_O_2_ catabolic processes. Besides the well-known role of hydrogen peroxide in microbial interactions [23,24], it is also one of the major metabolic by-products of many lactic acid bacteria [25], as these often lack respiratory chains and opt to reduce molecular oxygen to recycle NAD+ from NADH, with increased energetic yield as compared to the classical fermentation process. Analogous cases of hybrid metabolism have been recently reported by Marco et al. [26] in *Lactobacillus plantarum*, a microorganism with a pivotal role in fermented food production technology. Here, the authors described how combining features of respiration and fermentation would improve lactic acid bacteria function, thus enhancing product biosafety and quality [26]. The resistance of lactic bacteria to hydrogen peroxide is granted by the absence of oxidant-sensitive dehydratases and mononuclear Fe(II) enzymes [25,27]. Instead, the extensive involvement of the core microbiota in the biosynthesis of Fe(III)-chelating substances produced by aerobic or facultatively anaerobic bacteria (i.e., siderophores) suggests the activation of hybrid metabolism in the core microbial consortium, although tailored investigations would be necessary to confirm this unconventional metabolic route.

The core microbiota was also focused on biological processes linked to the development of the typical nutraceutical and gustatory characteristics of the dairy product, as was supported by the overall involvement of the microbiota in the biosynthesis and/or transformation of a variety of proteins, lipids, and amino acids. Specifically, the way in which bacterial activities related to lipid metabolism and fatty acid biosynthesis are linked to improved organoleptic properties in cheese and dairy products has already been described [28,29]. In addition, the microbiota involvement in the “arginine biosynthetic process via ornithine” indicated the continuous control exerted by the whole microbiota composition on the development of nutraceutical features, considering the role of ornithine in the production of bacteriocins and natural antibiotics. Additionally, arginine affects a variety of human physiological processes, such as growth/tissue repair, immune support, and cellular communications [30]. Moreover, the microbiota engagement in the 7,8-dihydroneopterin 3’-triphosphate biosynthetic process was suggestive of the production of B-group vitamins and folate, whose health-promoting effects range from anticarcinogenic activity to a reduced risk of cardiovascular diseases [31,32]. In line with biopterin production, the thiamine production further supported the beneficial effects exerted by the core-associated microbial community on the cheese organoleptic and health-promoting characteristics [33,34].

## 4. Materials and Methods

### 4.1. Cheese Samples and Experimental Design

The present work explored the microbial community associated with a typical raw goat milk cheese. Caprino Nicastrese cheese was employed as the study object, as an example of a traditional raw goat milk cheese. Following collection, the raw goat milk was coagulated for 60 min at 36 °C using 0.4 g/L goat rennet and without the addition of any starter culture. The resulting curd was manually cut into rice-sized pieces, shaped, and stored at room temperature for 48 h to drain out the residual whey. Cheese wheels were then salted for 24 h in brine with 30% (*w*/*v*) NaCl. Finally, the cheese was ripened in wooden axis in the storage basement of the cheese farms at 10–15 °C and 70–85% humidity.

**Figure 7:**
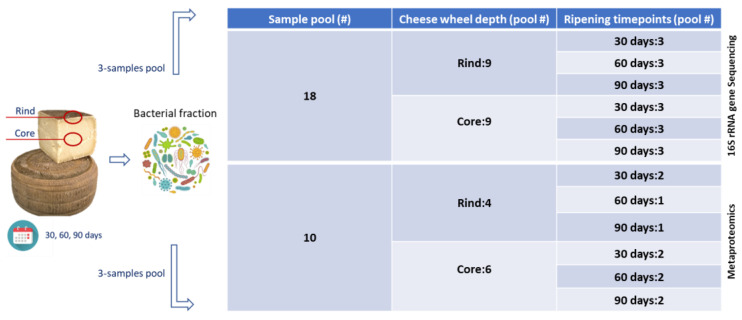
Experimental design and sampling strategy.

Samples from the surface and inner mass (i.e., rind and core, respectively) of the cheese wheels were aseptically collected with a sterile knife from 30-, 60-, and 90-day-ripened cheese wheels. Biological replicates were sampled as defined in Figure 7 and transported on ice to the laboratory for the subsequent isolation and analysis of the harbored microbiota.

Bacterial fractions were isolated from the rind and core of the cheese wheel at 30, 60, and 90 days of ripening. Pooling was performed, with each pool including bacterial extracts from three samples. Eighteen pools were subjected to 16S rRNA gene sequencing, 9 from the rind and 9 from the core. Each depth was composed of 3 sample pools taken from cheese wheels ripened for 30, 60, and 90 days. The metaproteomics survey relied on a total of 10 sample pools, 4 for the rind and 6 for the core depth. Two pools were considered from each ripening timepoint of the core depth, whereas two pools were excluded in the rind groups due to technical issues encountered during the analytical workflow. Specifically, one pool was omitted from the pool at 60 days and one from the group at 90 days of ripening.

### 4.2. Bacterial Fraction Enrichment

To avoid alterations to the microbiota composition and/or activity, all the steps of the bacterial fraction enrichment were performed at 4 °C, with the temperature kept under strict control. Briefly, independent aliquots of 0.5 g of each biological replicate per sample type were finely grated and homogenized with 15 mL buffer containing 50 mM Na_2_HPO_4_ and 0.1% Tween 80 at pH 8.0. Samples were then shaken on an orbital shaker at 1600 rpm for 10 min. Following this, the samples were centrifuged for 20 min at 2500× *g*. The supernatant was collected in a new tube and subjected to four more rounds of shaking/centrifuge/resuspension, whereas the pellet from each step was gently resuspended and collected in a single clean “pool vial”. The “pool vial” was finally centrifuged at 12,000× *g* for 20 min, resulting in the collection of a bacterial pellet from an original amount of 0.5g cheese aliquots [1,14,35].

The enriched bacterial fractions represented the common starting point for the two analytical approaches employed in the present study: 16S rRNA gene sequencing and metaproteomics (Figure 7).

### 4.3. 16S rRNA Gene Sequencing and Metataxonomic Analysis

#### DNA Extraction and Library Preparation

Cheese DNA was extracted from 9 rind and 9 core samples, respectively, 3 for each ripening time point (Figure. 7) according to the EZ1 DNA Tissue protocol (Qiagen, Hilden, Germany). Starting from 40 mg, 190 µL of buffer G2 and 10 µL of proteinase K solution were added to each sample aliquot, before incubation at 56 °C in an Eppendorf^®^ Thermomixer until complete sample lysis, with vortexing 2–3 times per hour to disperse the sample. Two hundred microliters of supernatant were transferred to a new 2 mL sample tube, and the automated EZ1 extraction was finalized. The amplification of the V3–V4 variable region from the bacterial 16S rRNA gene (∼460 bp) was carried out using the primers 16S_F 5′-(TCG TCG GCA GCG TCA GAT GTG TAT AAG AGA CAG CCT ACG GGN GGC WGC AG)-3′ and 16S_R 5′-(GTC TCG TGG GCT CGG AGA TGT GTA TAA GAG ACA GGA CTA CHV GGG TAT CTA ATC C)-3′, according to the MiSeq rRNA Amplicon Sequencing protocol (Illumina, San Diego, CA, USA). The PCR reactions were set up using 2 × KAPA Hifi HotStart ready Mix kits (KAPA Biosystems Inc., Wilmington, MA, USA). DNA amplicons were cleaned up using CleanNGS kit beads (CleanNA, Waddinxveen, The Netherlands). A second amplification step was performed to obtain a unique combination of Illumina Nextera XT dual indices for each sample. The final libraries were cleaned up using CleanNGS kit beads; quantified by a Quant-iT PicoGreen dsDNA Assay Kit (Thermo Fisher Scientific, Waltham, MA, USA); and normalized to 4 nM. To generate 250 × paired-end 2 bp length reads, normalized libraries were pooled together and run on the Illumina MiSeq platform, according to manufacturer’s specifications.

### 4.4. Biocomputational and Statistical Analysis for Cheese Microbiota Profile Analysis

QIIME2 was used to analyze the paired-end sequencing reads [36]. Quality control, denoising, chimera removal, trimming, and the construction of the amplicon sequence variant (ASV) table were performed by the means of DADA2, implemented as a plugin in QIIME2 [37]. The taxonomy was assigned using a Naive Bayes model pre-trained on SILVA through the QIIME2 plugin q2-feature classifiers [38]. Alpha and beta diversity were computed by skbio.diversity using analysis of variance (ANOVA) and permutational analysis of variance (PERMANOVA), respectively; the latter was applied on phylogenetically informed weighted and unweighted UniFrac and Bray–Curtis distance matrices [39] with 9999 permutations to perform a paired comparison of the rind and core samples at different timepoints. Principal coordinate analysis (PCoA) plots were used to illustrate the beta diversity of samples. The ASV table was normalized using the cumulative sum scaling (CSS) method [40]; hence, the Kruskal–Wallis test was applied to compare taxonomic differences at the phylum (L2), family (L5), and genus (L6) levels. Python 3.7 was used to perform ecological statistical analyses. Three different levels of statistical significance were identified based on different *p* values (*p* ≤  0.001) and false-discovery rate (FDR) thresholds (*p* ≤  0.05, *p* ≤ 0.001) [41]. Phylogenetic Investigation of Communities by Reconstruction of Unobserved States (PICRUSt) [42], employing the Kyoto Encyclopedia of Genes and Genomes (KEGG) orthologs (KO) database, was used to determine ASVs and their microbiome functions. In addition, LEfSe (linear discriminant analysis effect size) was independently used to determine the features most likely to explain the differences between the rind and core of the cheese wheel at 30, 60, and 90 days of ripening.

### 4.5. Metaproteome Extraction and Quantification

Bacterial pellets obtained via the above-described bacterial fraction enrichment protocol were resuspended in protein extraction buffer (7M UREA, 2M Thiourea, 4% CHAPS) and subjected to 6 cycles of 1 min bead beating (Minilys, Bertin Tecnologies, Montigny-le-Bretonneux, France), interspersed with 1 min resting on ice. Bead-beating steps were performed by shaking each sample at 4000 rpm with an equal amount (1:1 *v/w*) of 0.1 mm zirconium silica beads. Following bead beating, the samples were heated up to 60 °C for 10 min and centrifuged for 20 min at 12,000× *g* and 4 °C. The supernatant containing the extracted metaproteome was collected in a clean tube and further processed for the metaproteomic analytical workflow.

Extracted proteins were quantified using Bio-Rad Protein Assay Dye Reagent Concentrate (Bio-Rad, Hercules, CA, USA) following the manufacturer’s instructions. Approximately 50 μg of the extracted proteins was precipitated by incubation (30 min at 4 °C) with precooled 20% trichloroacetic acid (TCA) and kept for further processing.

### 4.6. Trypsin Digestion and Mass Spectrometry Analysis

Precipitated proteins were digested in solution. Briefly, 50 μg of total proteins for each sample was treated for disulfide bond reduction with 10 mM DTT for 1 h at +37 °C and alkylated with 20 mM IAA at +37 °C for 1 h in the dark. Iodoacetamide excess was removed by the incubation of the sample with 1.61 mM DTT at +37 °C for 20 min. Sample digestion was carried out overnight at +37° C using trypsin in a 1: 50 (*w*/*w*) ratio with respect to the protein content. Enzymatic digestion was stopped by the addition of 0.1% FA (*v*/*v*). Tryptic peptides were purified and desalted using self-assembled C18 Stage Tips [43]. Tips containing the C18 membranes with the bounded peptide mixture were eluted with 5% acetonitrile (5% ACN/0.1% TFA), dried in the vacuum centrifuge, and stored at −20 °C until mass spectrometry measurements.

Prior to MS/MS measurement, the dried peptide mixture was suspended in 0.1% FA and loaded onto a precolumn Acclaim PepMap100 C18 (5 μm, 100 Å, 300 μm i.d. × 5 mm) (Thermo Scientific, San Jose, CA, USA). Following 5 min of trapping, operating at 10 μL/min in eluent A, peptides were separated by an Easy-Spray PepMap C18 column (2 µm 100 Å 15 cm × 50 µm ID) with a Thermo Scientific Dionex UltiMate 3000 RSLC nano system (Sunnyvale, CA, USA).

Analyses were performed using an aqueous solution of FA (0.1%, *v/v*) as eluent A and ACN/FA (99.9:0.1, *v*/*v*) as eluent B in the following gradient elution: (i) 5% of eluent B (7 min); (ii) from 5 to 35% of eluent B (113 min); (iii) from 35 to 99% of B (15 min); (iv) 99% of B (10 min); (v) from 99 to 5% of B (2 min); (vi) 5% of B for column conditioning (13 min). The column was kept at 35 °C and operated at a flow rate of 300 nL/min; the injection volume was set at 5.0 μL.

Peptides were directly eluted into Orbitrap Elite nanoESI-MS/MS (Thermo Fisher Scientific, Waltham, MA, USA). Tandem mass spectrometry measurements were performed in positive full-scan acquisition mode in the 350–2000 m/z range and with a resolution power of 60,000. The nanoESI tuning parameters were as follows: capillary temperature 250 °C, source voltage 1.5 kV, sheath gas 0, auxiliary gas 0, and S-lens RR level 50%. MS/MS analyses were performed in data-dependent scan (DDS) mode by selecting and fragmenting the twenty most intense multiple-charged ions of the collected full-scan spectra using collision-induced dissociation (CID, 35% normalized collision energy) with a resolution power of 60,000. Only precursors with a charge state of 2–7 and an intensity above the threshold of 5 × 103 were collected for MS/MS. The DDS parameters were set as follows: exclusion mass width relative reference mass in the low and high range 10 ppm, minimum signal threshold (counts) 500, default charge state 2, activation time 10 ms [44].

### 4.7. Bioinformatics Data Analysis and Data Integration

#### 4.7.1. Protein Identification and Quantification

MS raw spectra were processed through Proteome Discoverer and MaxQuant software following a two-step database-dependent search (DDS) approach, as reported previously [15]. Briefly, raw files were first processed by Thermo Proteome Discoverer software (v.2.2) and searched against the UniProt KB bacteria database. Methionine oxidation was set as the variable modification and the carbamidomethylation of cysteine as the fixed modification. The SequestHT node thresholds were set to “automatic”, and a filter considering only entries with at least one peptide per protein was chosen. All other filters and settings of the software were kept as default, including protein grouping with peptide confidence set as “high” and a delta Cn of 0.1. The percolator node supporting a strict maximum parsimony principle was activated with a false discovery rate of 1%.

The first DDS enabled the assessment of the microbial community composition at the family level, leading to the construction of a smaller in-house database accounting for the bacterial families identified in both the metaproteomics and 16S rRNA gene-sequencing investigations. The customized database was employed in the second DDS of the MS raw data performed on MaxQuant (v 1.6.17.0) set to LFQ modality for peptide identification and protein inference and quantification. Cysteine carbamidomethylation was set as the fixed modification and methionine oxidation as the variable modification. Two missed cleavage sites were allowed for in-silico protease digestion, and peptides had to be fully tryptic. All other parameters of the software were set as default, including peptide and protein FDR < 1%, at least 1 peptide per protein, precursor mass tolerance of 4.5 ppm after mass recalibration, and a fragment ion mass tolerance of 20 ppm. The mass spectrometry proteomics data were deposited into the ProteomeXchange Consortium via the PRIDE partner repository with the dataset identifier PXD032280.

#### 4.7.2. Ecological and Functional Characterization of Microbiota by Metaproteomics

Information on the taxonomic composition of the microbiota, as assessed by the identified protein repertoire, was gathered from the protein annotation of the UniProt KB database, whereas the quantitative microbiota composition was determined based on the LFQ intensities relative to each bacterial member on a family basis. The logarithmic transformation of the cumulative intensities on a family basis was accomplished while comparing the microbiota composition in the diverse sample groups.

Identified protein repertoires were functionally categorized into biological processes and molecular functions according to the Gene Ontology (GO) data repository. Abundance profiles of the identified proteins (LFQ values) were subjected to statistical investigation using Primer7 v.7 statistical software (PRIMER-E, Plymouth, UK). Principal component analysis (PCO) was conducted based on the square root transformation of the protein LFQs. Statistical differences across the samples were calculated by performing ANOVA and PERMANOVA. Parametric T-tests assessing the discriminating role of the bacterial families in the microbiota composition were conducted, and the results were visualized in iMetalab using shiny apps (https://shiny.imetalab.ca/). Linear discriminant analysis effect size (LEfSe) was calculated using the galaxy platform (https://usegalaxy.org). Heat maps visualizing microbial community composition across the samples and the functional classification of the identified proteins were drawn using heatmap.2, provided by the gplots package implemented in R v.4.2.0 software (http://www.R-project.org). Correlation analysis was performed through the corrplot package implemented in R v.4.2.0 software (http://www.R-project.org).

## 5. Conclusions

This is the first metaomics-based study of a typical raw goat milk cheese. Goat cheeses are commonly consumed and popular for their gustatory properties and health benefits. The combination of 16S rRNA gene sequencing and the metaproteomic approach enabled the in-depth characterization of the composition and activity of the microbiota at different cheese-wheel depths and provided insights into the structural dynamics of the microbial community during ripening. Altogether, this explorative study provided basic knowledge on the microbial community harbored in this fascinating dairy product and offered suggestions for further objective-tailored research lines. The biological functions expressed by the investigated microbiota are certainly of interest in the context of the biological safety of traditional products, including the development of strategies and precautions to keep the risk of zoonoses and/or foodborne diseases to a minimum. In addition, understanding the contribution of the microbiological footprint to the development of the flavor and texture of this cheese could greatly influence cheesemaking technology by informing microbiota modulation practices aimed at amending the quality and standardization of typical dairy products.

## Data Availability

The datasets supporting the conclusions of this article are available in the PRIDE repository (PXD032280; https://www.ebi.ac.uk/pride/archive/).

## Supplementary Materials

The following supporting information can be downloaded at: https://www.mdpi.com/article/10.3390/ijms232214131/s1. Additional File S1. Good’s coverage of the 16S rRNA sequencing data. Box plot in the panel A shows the distrubution of the Good’s indexes of the sequencing data in the rind samples. B-panel displays the distribution according to the cheese wheel depth and ripening timepoints. Additional File S2. Metabolic potential of the microbial communities. The functional potential of the metabolic community is predited via PICRUST analysis of the microbiota harbured on the rind and core (upper stacks) and in the micrbiota of the rind and core at hte ripening timepoints of 30, 60 and 90 days (lower stacks). Additional File S3. Functional dataset visualization. PCO plot are relative to the samples stratified according to cheese wheel depths (A-panel) and cheese wheel ad ripening timepoints (B-panel). Additional File S4. Microbial community richness and evenness indexes. Additional File S5. Linear Discriminant Analysis among the bacterial families of rind and core. The cladogram summarizes the LDA analysis of the identified proteins grouped for the bacterial families they belong to and sorted on a cheese wheel depth basis. Log10 LDA score depicted in the bar-chart below indicated the bacterial families significantly overrepresented in the Core and Rind microbial consortia as of the LDA analysis. Additional File S6. Identified metaproteome dataset and functional annotation into the major data repositories for the protein ontology. Additional File S7. Functional statistics of the identified proteins. Identified proteins are functionally categorized. Cumulative abundance of each functional class is employed for the statistical evaluation of the microbial communities on a functional view. The sole rind versus core comparison scored a statistically significant difference (*p* < 0.01). A- panel visualizes the samples hierarchical clustering. B-panel depicts the dataset on a PCO plot. Panel C represent the same scattering of the dataset by labelling the samples according to the ripening timepoints (*p* > 0.05).
