# Supplementary figures and images for: The Functional Characteristics of Goat Cheese Microbiota from a One-Health Perspective
Source: Int J Mol Sci. 2022 Nov 16;23(22):14131. doi: 10.3390/ijms232214131 (PMC9698706; doi:10.3390/ijms232214131)

## Slide 1
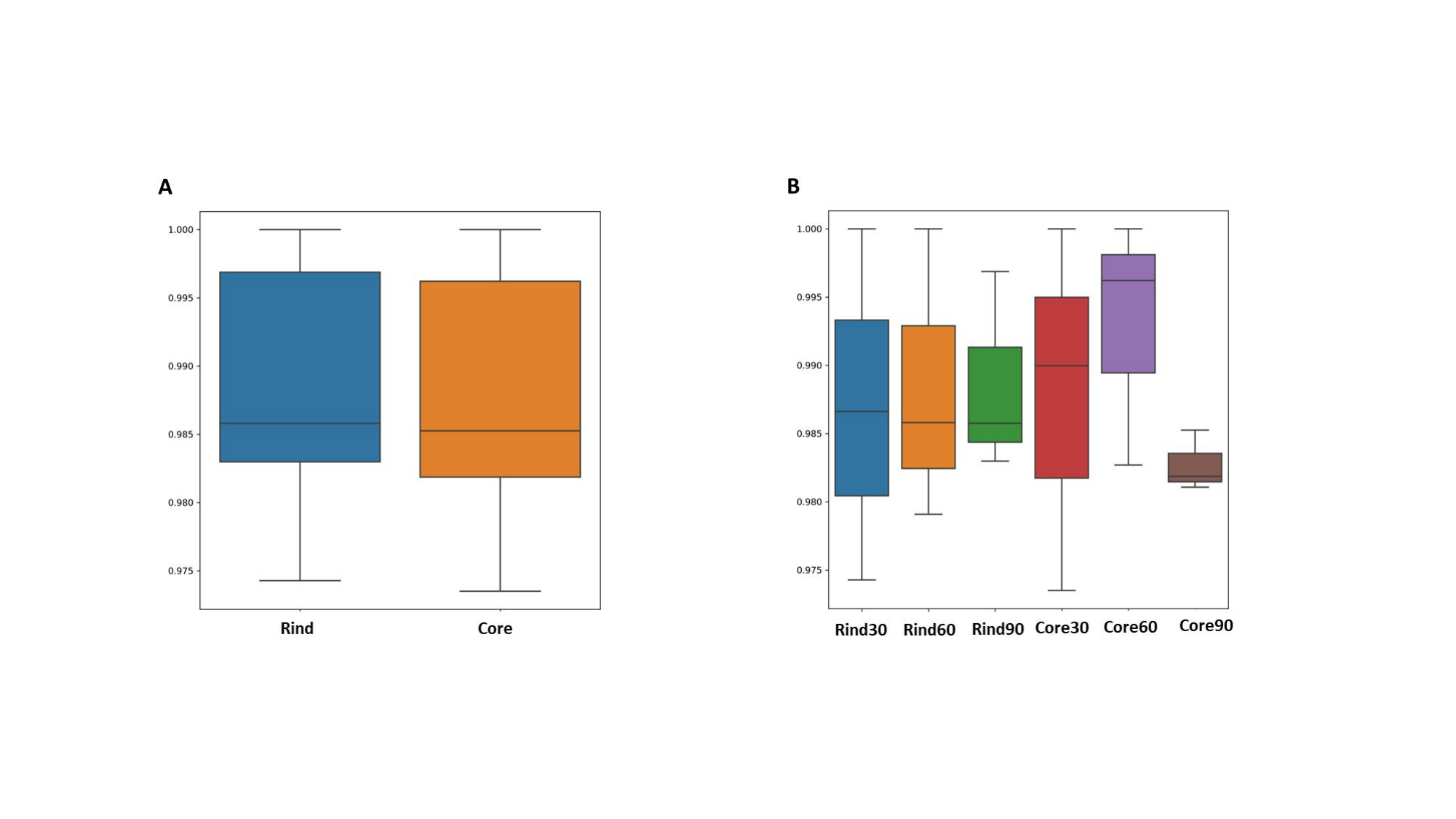

Supplement: Supplementary file 1 [file ijms-23-14131-s001.zip › Additional File S1.pptx]

## Slide 1
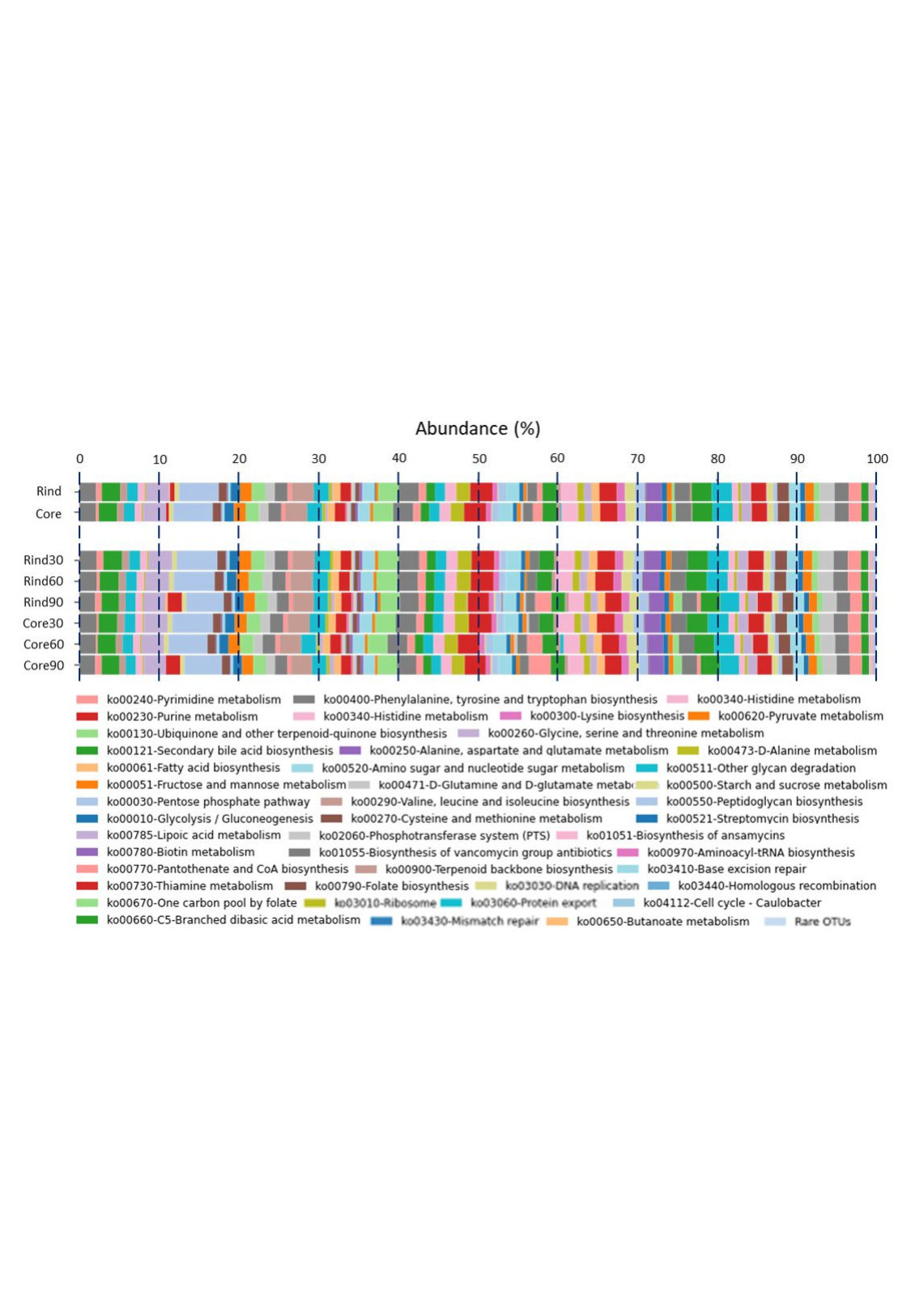

Supplement: Supplementary file 1 [file ijms-23-14131-s001.zip › Additional FIle S2.pptx]

## Slide 1
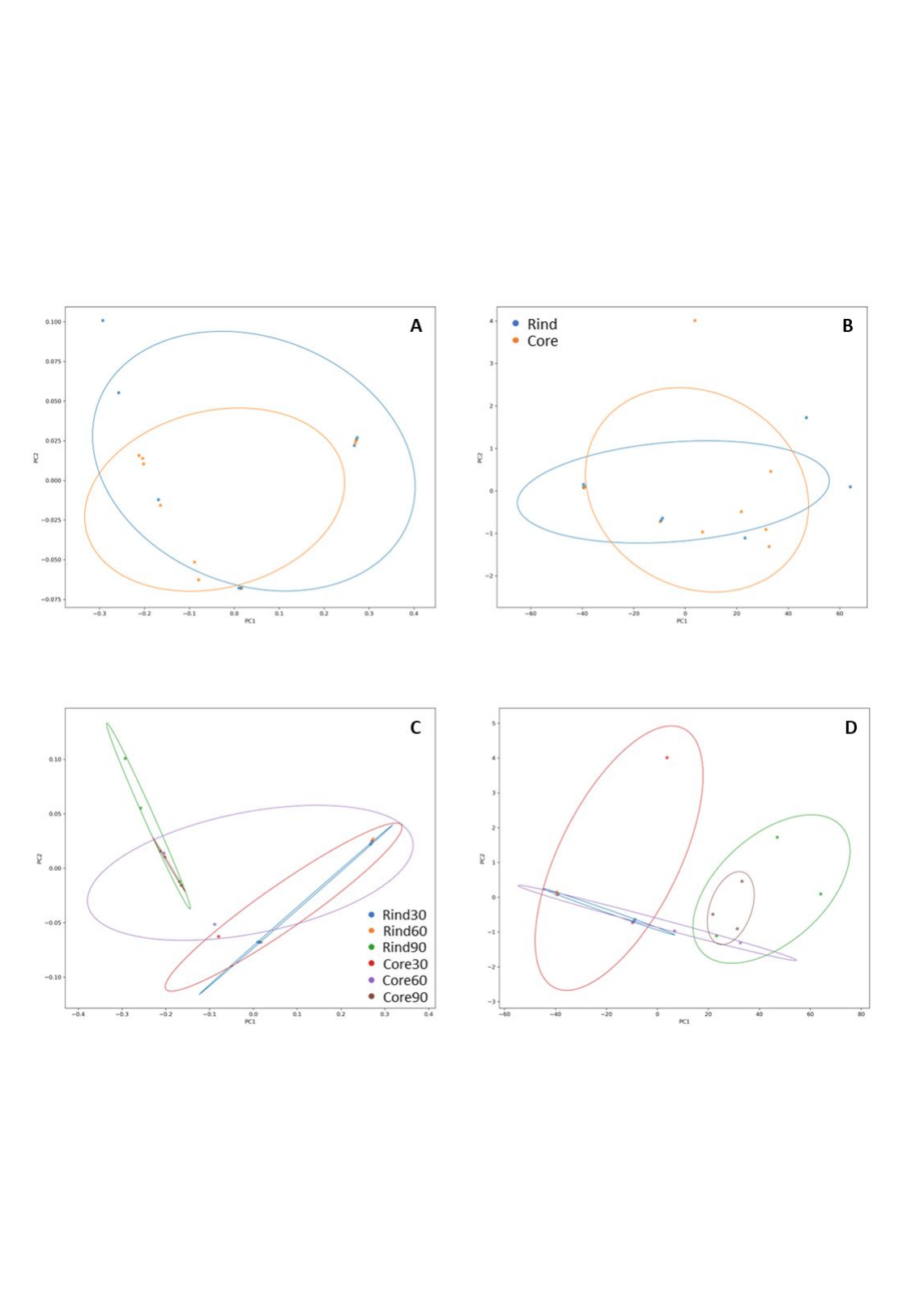

Supplement: Supplementary file 1 [file ijms-23-14131-s001.zip › Additional File S3.pptx]

## Slide 1
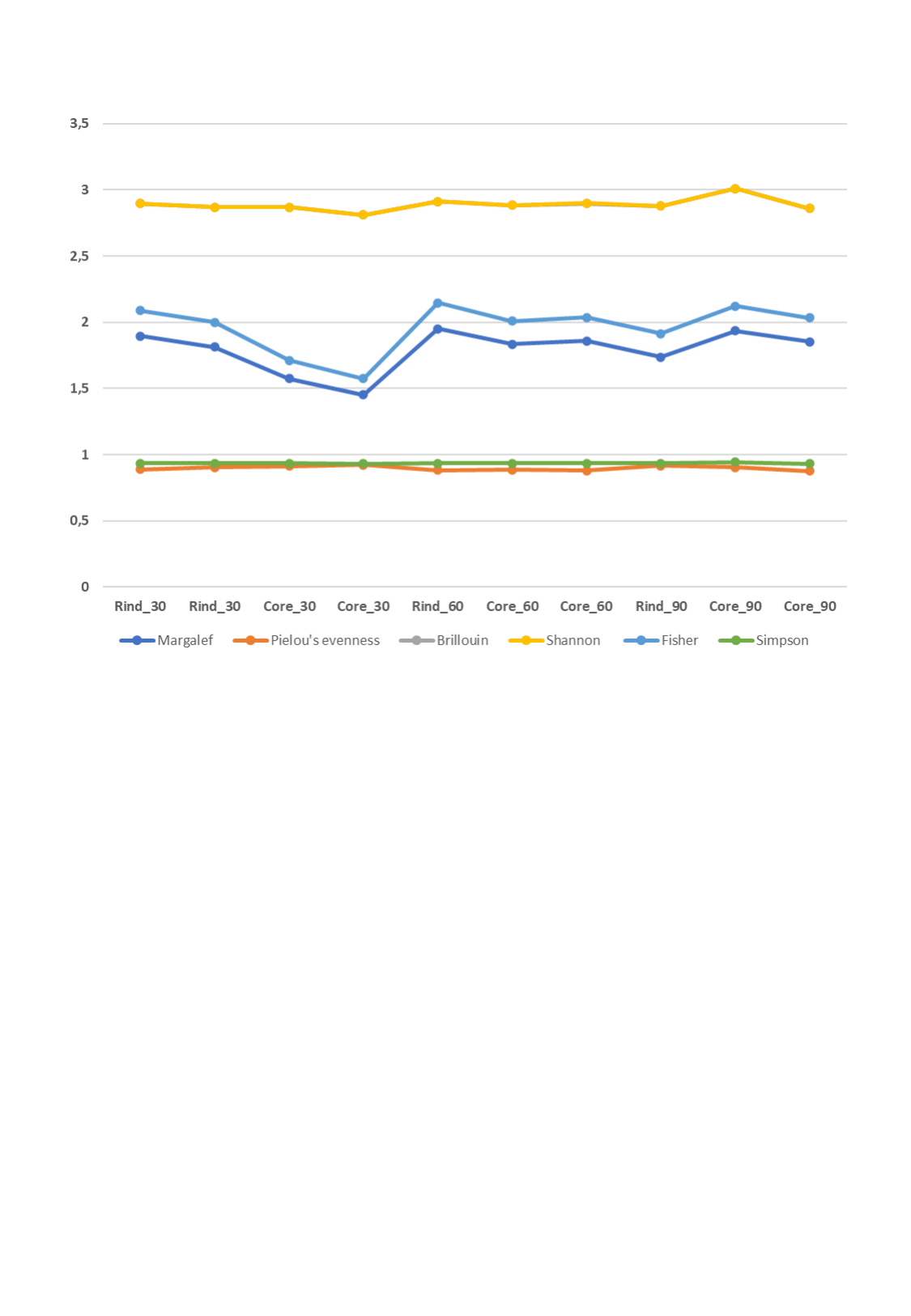

Supplement: Supplementary file 1 [file ijms-23-14131-s001.zip › Additional File S4.pptx]

## Slide 1
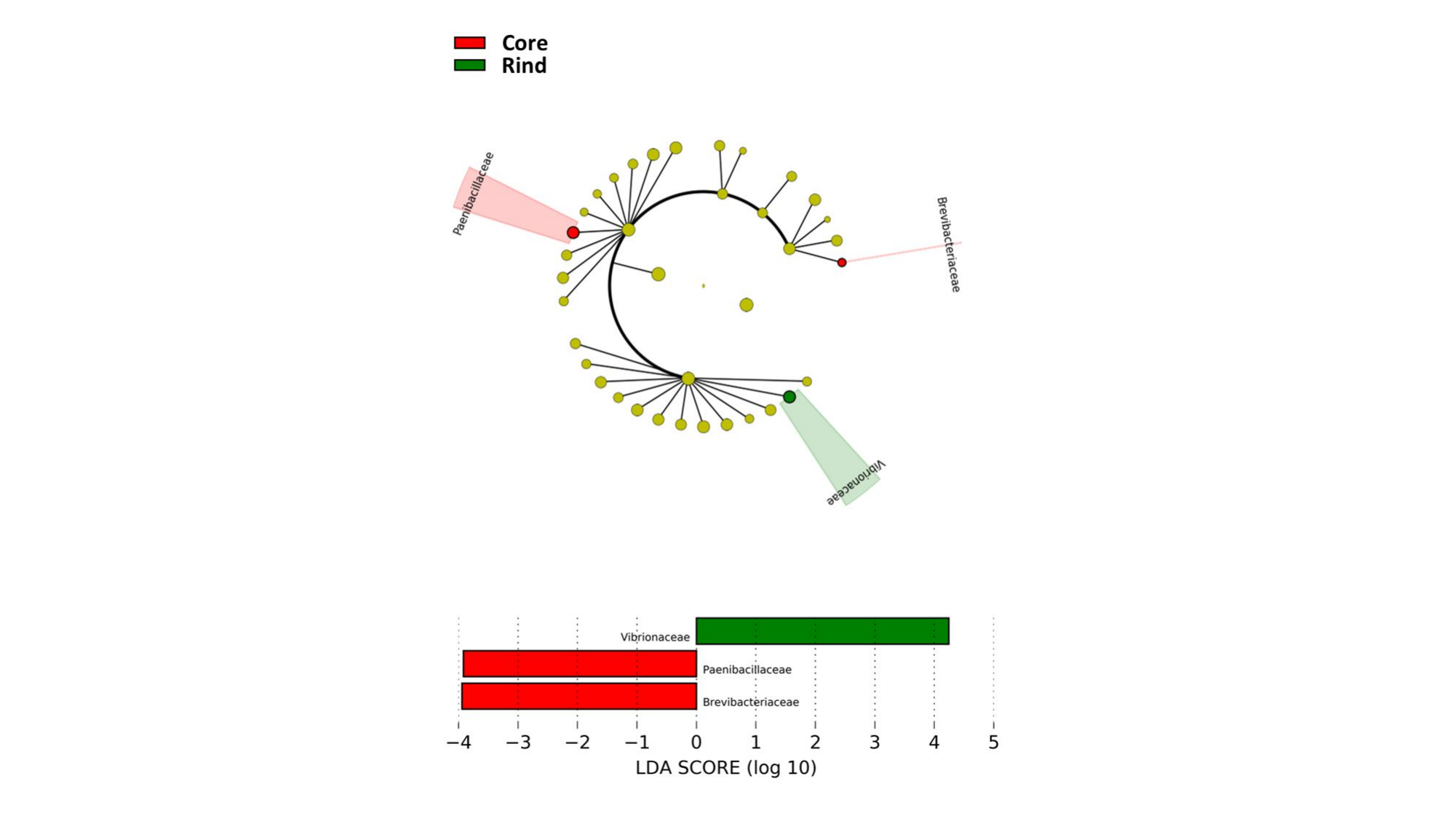

Supplement: Supplementary file 1 [file ijms-23-14131-s001.zip › Additional File S5.pptx]

## Slide 1
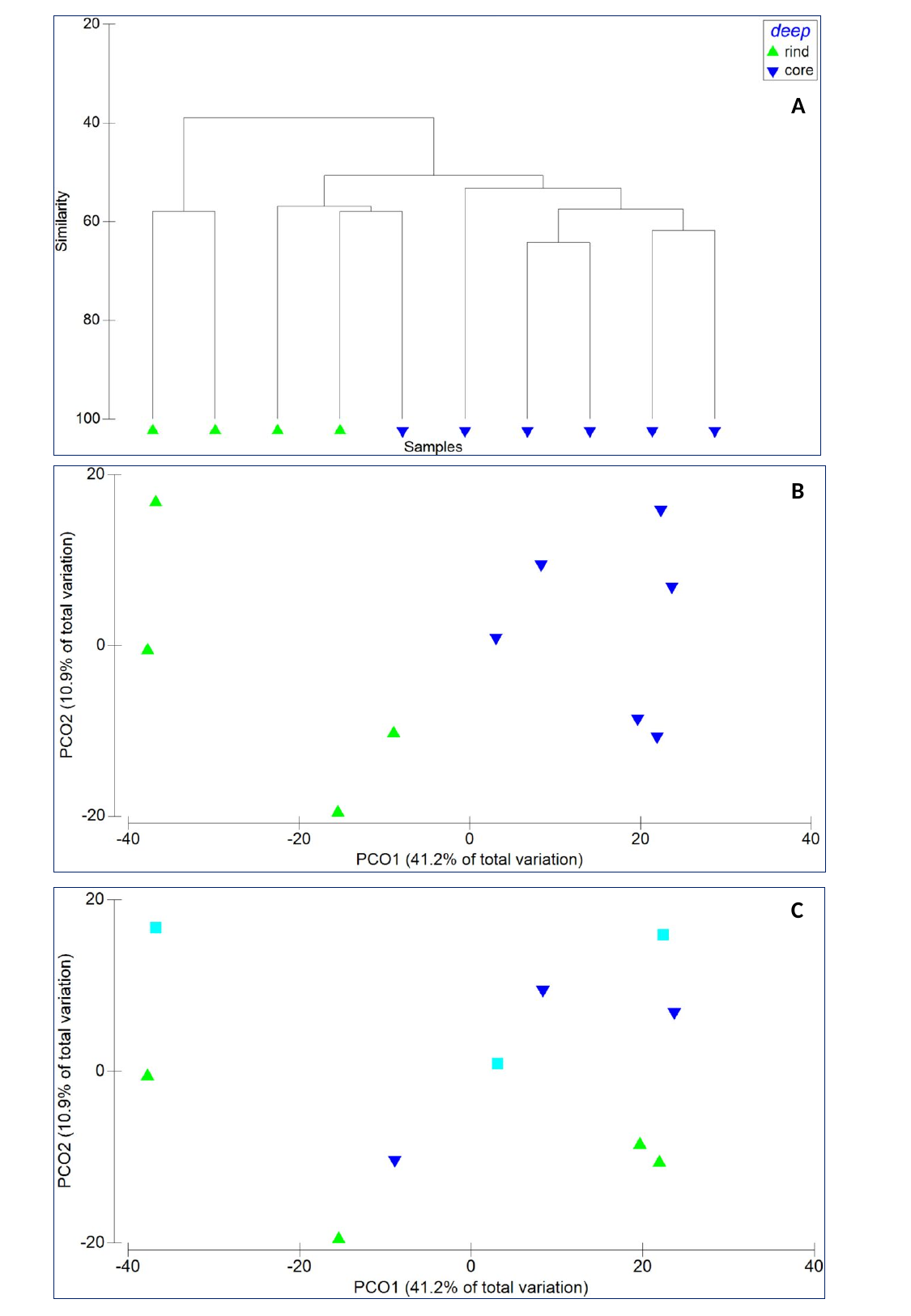

A
B
C

## Slide 2
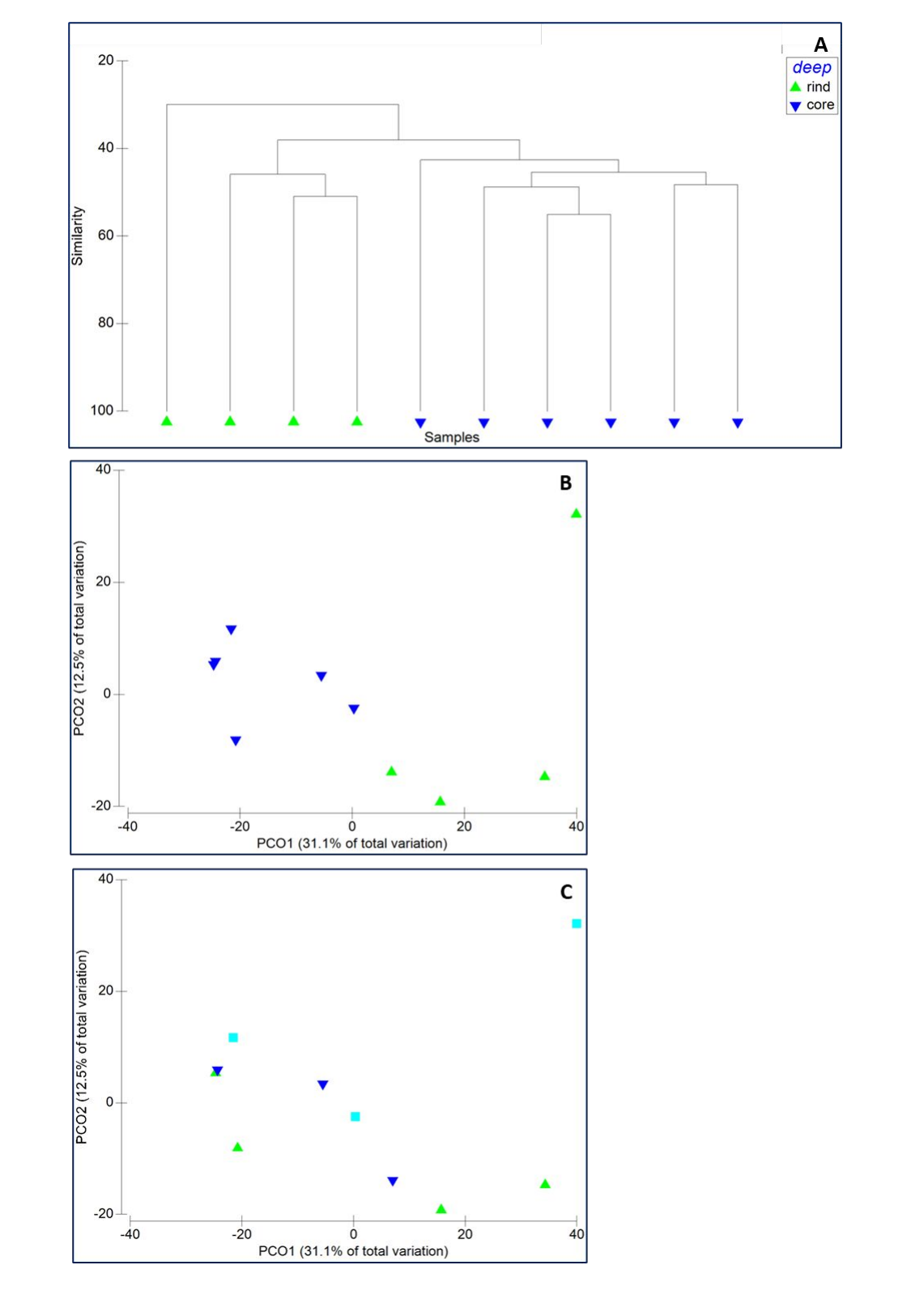

## Slide 3
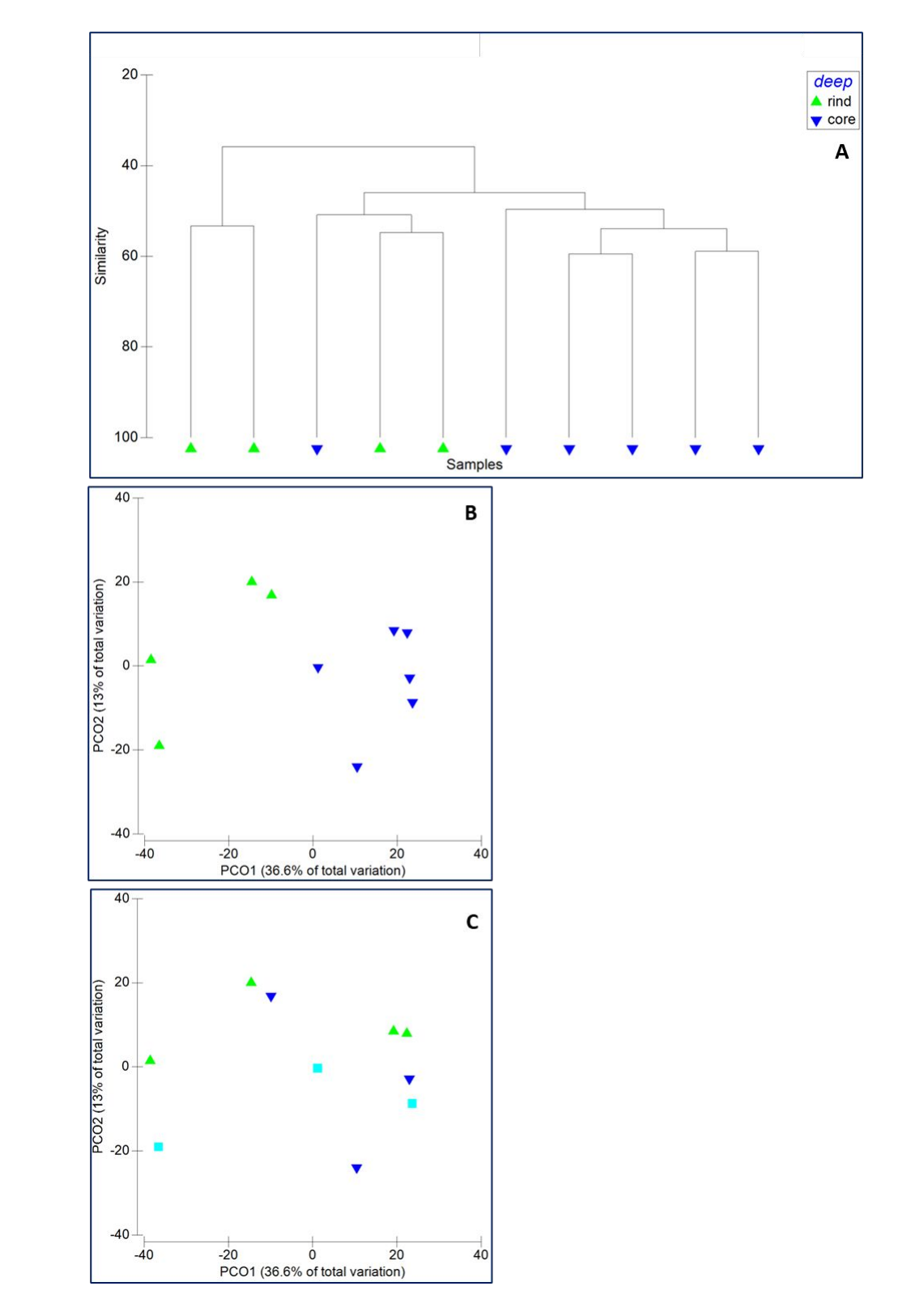

## Slide 4
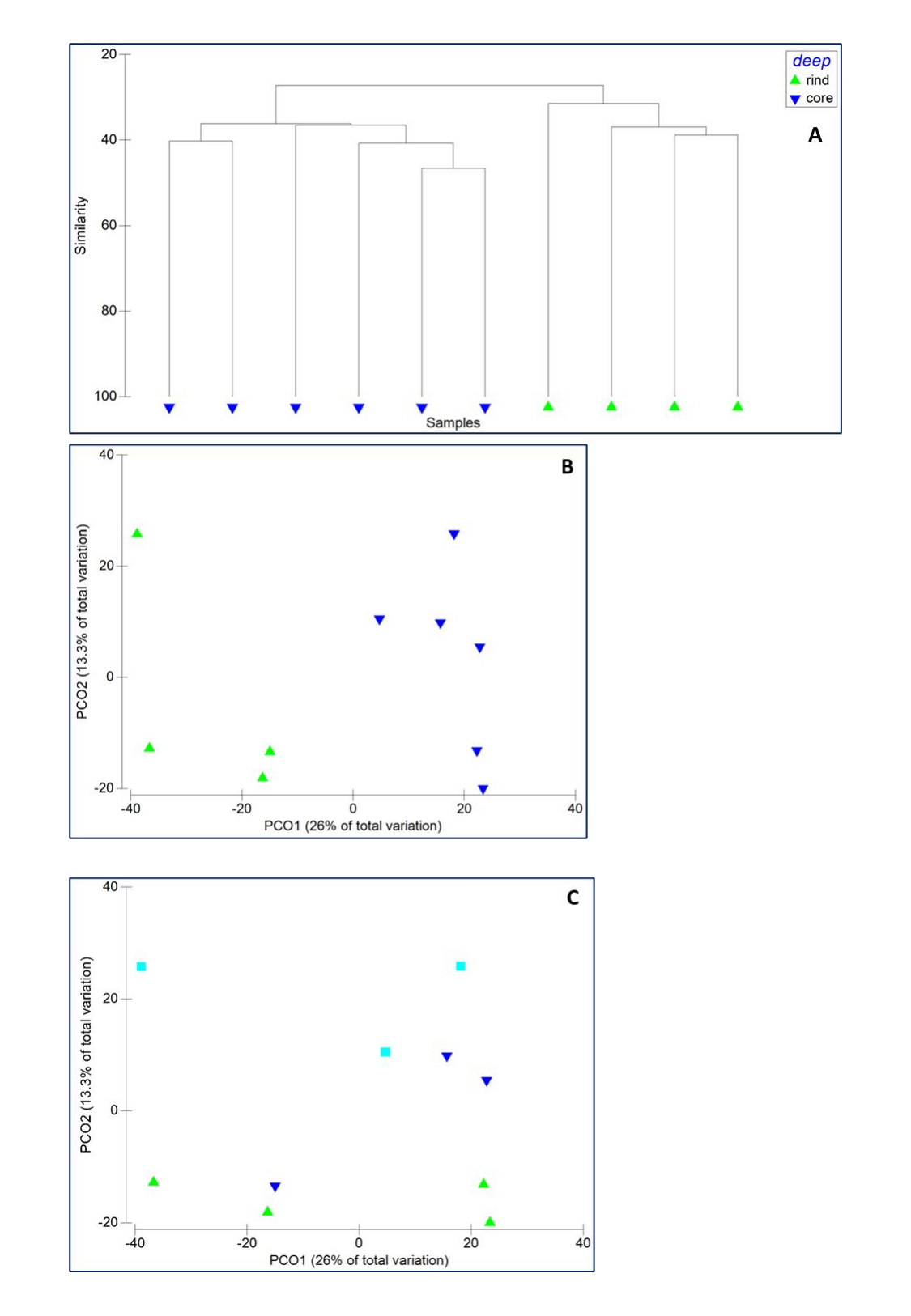

## Slide 5
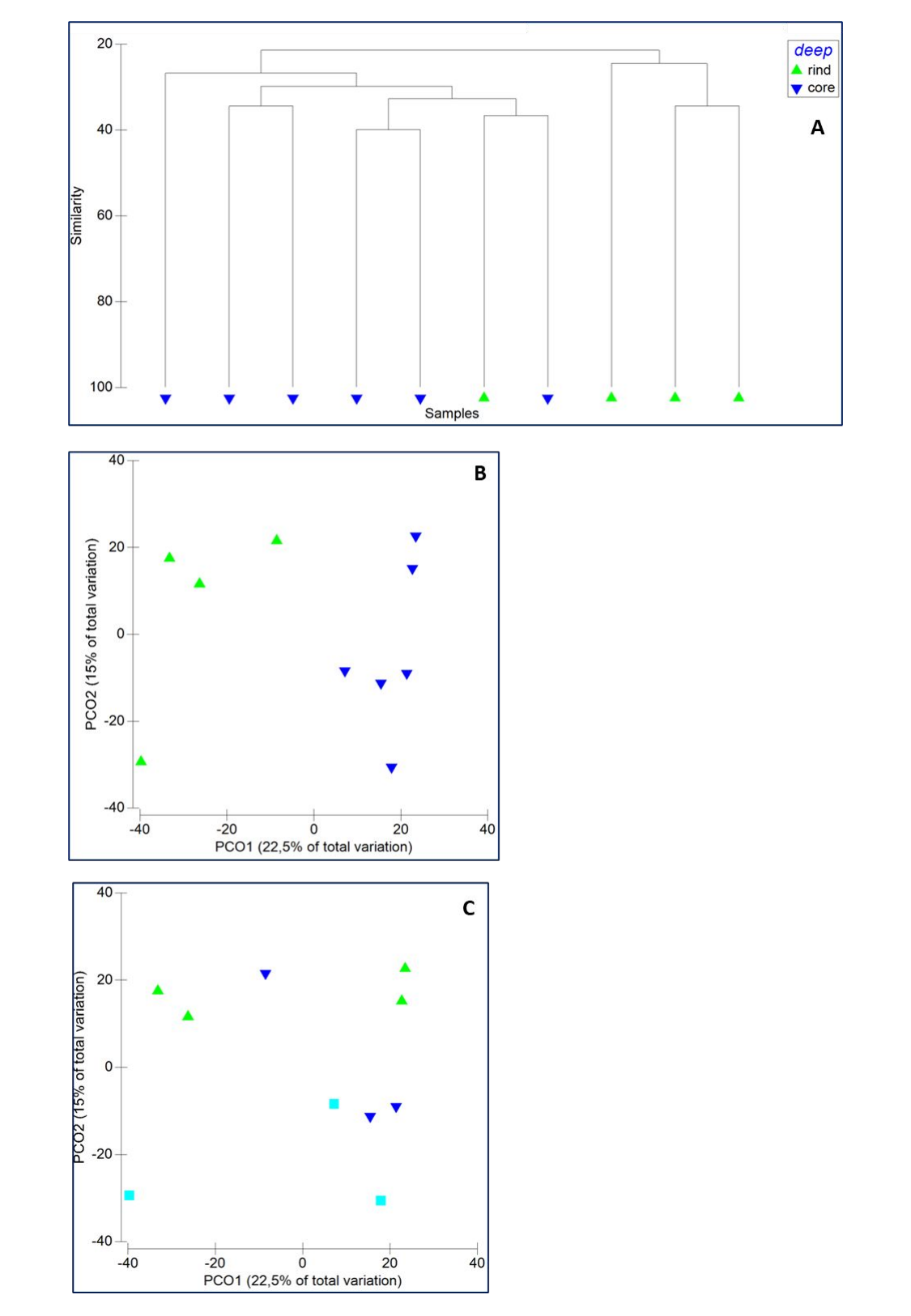

Supplement: Supplementary file 1 [file ijms-23-14131-s001.zip › Additional File S7.pptx]
